# A rare presentation of orbital spindle cell carcinoma a case report and brief review of the literature

**DOI:** 10.1186/s12886-023-03125-7

**Published:** 2023-09-08

**Authors:** Mansooreh Jamshidian Tehrani, Ali Rashidinia, Fahimeh Asadi Amoli, Amirreza Esfandiari

**Affiliations:** Department of Ophthalmology, Farabi Eye Hospital, Tehran University of Medical Science, Tehran, Iran

**Keywords:** Spindle cell carcinoma, SCC, Limbal ischemia

## Abstract

**Background:**

To describe a case of orbital spindle cell carcinoma which presented with limbal ischemia and briefly review the literature.

**Methods:**

Retrospective case report and brief literature review.

**Results:**

A 61-year old man presented with blepharoptosis, periorbital pain, decreased vision and limbal ischemia. He did not mention any previous illness and did not take any kind of drugs. Imaging revealed an orbital mass that was positive for SMA, Vimentin and CD99 and negative for S100. We treated the patient with chemotherapy and followed him for other complications that occurred throughout disease course.

**Conclusion:**

Spindle cell carcinomas are a rare variant of squamous cell carcinoma (SCC) with dual malignant histologic differentiation of squamous and mesenchymal cells. Few cases of orbital spindle cell carcinoma have been reported, which have been either secondary to distant metastasis or regional spread. In this study, we have reported the first case of primary orbital spindle cell carcinoma presenting with limbal ischemia. Further studies are needed to describe the different clinical presentations and management strategies of this rare clinical entity.

## Background

Spindle cell carcinoma, also known as carcinosarcoma or sarcomatoid carcinoma, is a rare variant of squamous cell carcinoma (SCC) characterized by dual malignant histologic differentiation of squamous and mesenchymal cells [1, 2]. Some studies have suggested that spindle cell carcinoma is more aggressive than typical SCC, with distant metastasis being more common at presentation [3].

Periocular spindle cell carcinoma is a rare occurrence, with most cases involving the conjunctiva and/or cornea [4–6]. Orbital involvement is even rarer, and cases have been reported secondary to distant metastases or local invasion from adjacent structures [7–11]. Due to the small number of reported cases, periocular behavior of these tumors is mostly unknown.

In this study, we report the first case of primary orbital spindle cell carcinoma presenting with limbal ischemia. We also briefly review the current literature on this rare tumor.

## Case presentation

**Fig. 1:**
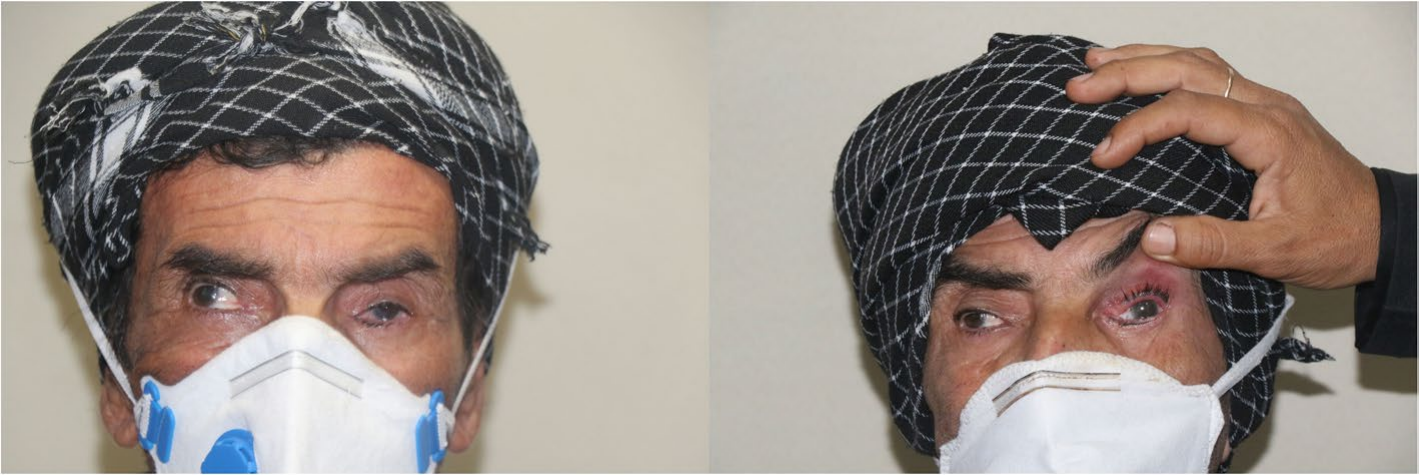
External photograph of the patient before (left) and after (right) operation. Notice the left ptosis and exotropia on the left

**Fig. 2:**
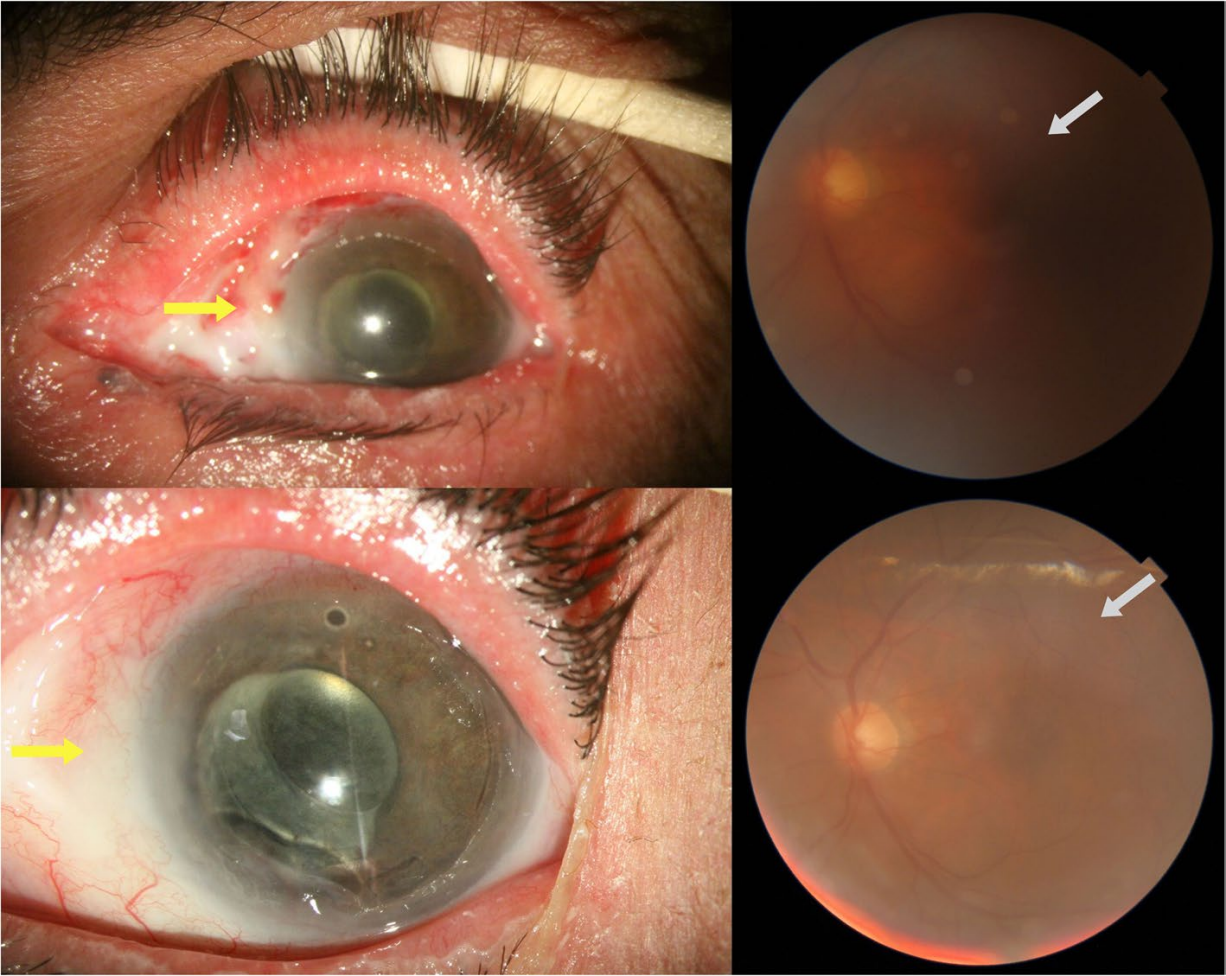
Slit photo and Fundus photo of the left eye before (top) and after (bottom) operation. Note improvement of limbal ischemia (yellow arrows) and vitritis (white arrows)

**Fig. 3:**
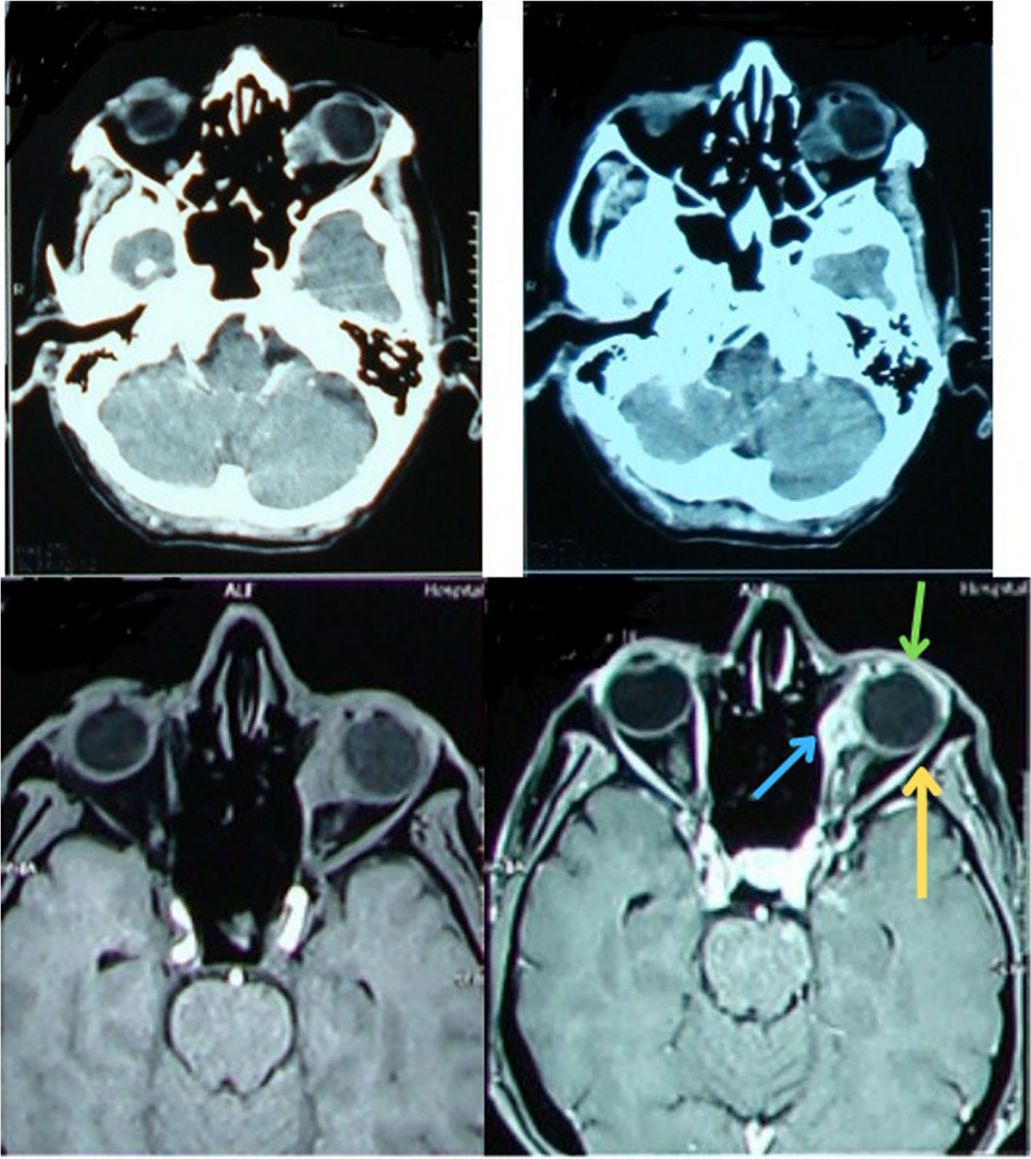
CT scan & MRI of patient revealing left intraconal mass before and after operation (Top right-CT scan before operation. Top left-CT scan after operation. Bottom right-T1 MRI before operation (Note the Lateral recuts: yellow arrow, Medial recuts and tumor compressing the globe: blue arrow, globe: green arrow). Bottom left-T2 MRI before operation). Because of the diagnostic nature of the operation the size of the tumor is not changed significantly

**Fig. 4:**
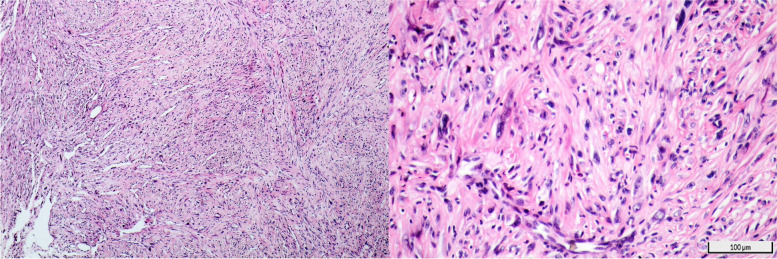
Biopsy sections of the mass show hypercellular spindle cell neoplasm with cellular atypia and some mitotic features. H& E staining, right: low (*10) and left: high (*40) magnification. Note size scales

A 61-year-old man presented to our general ophthalmology clinic at “Imam Khomeini hospital complex (IKHC)” with left blepharoptosis and periorbital pain of 2 months duration. His past medical history was unremarkable, and he denied using any medications or illicit drugs. His past ocular history included uncomplicated cataract surgery of the left eye 4 years before presentation. He denied any past ocular trauma, chemical or thermal burn. A comprehensive ophthalmic examination was performed. The best corrected visual acuity (BCVA) was 20/22 in either eye. External examination revealed mild eyelid swelling and moderate blepharoptosis of the left eye. Enophthalmos of the left eye was also noted. Hertel exophthalmometry was performed which revealed 2 mm of left enophthalmos. On ocular motility examination, moderate limitation of movement of the left eye was noted in adduction and downgaze, along with minimal limitation of abduction and normal upgaze, and there was almost 30 prism left exotropia. Further examination was performed using slit lamp biomicroscopy. Anterior segment examination revealed 300 degrees of limbal ischemia. The cornea was clear and no epithelial defect was noted. Conjunctival injection was noted. The patient was pseudophakic and the intraocular lens was well in place with no significant posterior capsular opacity. The rest of the anterior segment examination was within normal limits. A posterior segment examination using a + 90 diopter lens was performed, which only revealed a blunted foveal reflex. Examination of the right eye was normal, apart from a blunted foveal reflex (Figs. 1 and 2). Spiral orbital computed tomography (CT) revealed an intraconal mass so for further evaluation of the mass we performed Magnetic resonance imaging (MRI) which confirmed the mass involving the left sclera and tenon (Fig. 3). We decided to perform a transconjunctival orbitotomy to biopsy the lesion using a superotemporal approach, in the operating room because the mass was infiltrative and not circumscribed by capsule we performed a diagnostic biopsy (debulking the tumor as much as possible) and the specimen was sent for histopathological analysis. Histopathology revealed nests of squamous and spindle cells (mostly spindle) with cellular atypia, abundant mitotic figures and foci of necrosis (Fig. 4). Immunohistochemistry was also performed which was positive for smooth muscle antigen (SMA), vimentin and CD 99 but negative for S100. General physical examination and further evaluation with positron emission tomography and abdominopelvic CT revealed no other neoplastic lesions. A diagnosis of primary spindle cell sarcoma of the orbit was made and the patient was referred to “Imam Khomeini hospital complex (IKHC)”. The patient underwent 6 cycles of chemotherapy using the AIM regimen consisting of 50 mg of doxorubicin and 2 g of ifosfamide in the first 3 days and 600 mg of MESNA in the next 5 days. The interval between each cycle was 3 weeks. The patient was followed 9 months later. He complained of a recent reduction of vision in the left eye of almost 2 months duration, along with photophobia. A comprehensive ophthalmic examination was performed once again. BCVA was 20/22 and 20/200 in the right eye and the left eye, respectively. Examination of the right eye was within normal limits. External examination of the left eye revealed blepharoptosis of the left eye. No swelling was noted in the periocular region. Hertel exophthalmometry revealed less than 1 mm of left enophthalmos. On ocular motility examination, 30 prism diopters of exotropia were noted in the left eye, with severe limitation of movement in adduction and upgaze and mild limitation in downgaze and normal abduction. Slit lamp biomicroscopy revealed a corneal stromal opacity in the inferonasal quadrant without any corneal epithelial defect. No limbal ischemia was noted. The rest of the anterior segment examination was normal. Posterior segment examination revealed moderate vitritis and vitreous haze along with inferior macula-sparing retinal detachment with serous subretinal fluid and areas of pigment epithelial change in a leopard pattern suggesting chronicity. No retinal break was found. Fluorescein angiography revealed early hyperfluorescence secondary mostly to leakage, with later pooling and leopard pattern of hyperfluorescence. A decision was made to perform another imaging procedure. MRI of orbit and brain with and without contrast was done and there was no evidence of any abnormal lesion in the brain, on the other hand in orbit there was 17*10*9mm left intraconal mass, abnormally enhancing, hypointense in T1 and hyperintense in T2 medial to the left globe with involvement of medial rectus and globe (Fig. 1). A diagnosis of exudative retinal detachment secondary to the orbital mass was made. A decision was made to closely follow the patient and treat the underlying cause of the detachment. The patient was referred for further evaluation and management by the oncologist.

Our patient then lost follow-up for about 6 months and after that, we found that he was diagnosed with gastric adenocarcinoma with liver metastasis and was treated with chemotherapy which has not been completed at the time of writing the manuscript. The point to notice here is that it is not clear to us whether this GI tumor is related to the patient’s orbital tumor or not.

## Discussion and conclusions

Spindle cell carcinomas are considered a rare variant of SCC, with a predilection for the upper respiratory and digestive tracts [3]. Most studies have found a high male preponderance, and have suggested that spindle cell carcinomas behave more aggressively than their SCC counterparts [12]. However, other studies have suggested that tumor behavior and metastatic potential depend on the site of origin [13]. In addition, overall prognosis is often poor [2, 14].

Most primary spindle cell carcinomas of the periocular region arise from the conjunctiva [6, 15, 16]. There have been very few reports of spindle cell carcinoma of the orbit. Moreover, all known cases have been either distant metastases of other primary sources or secondary to regional spread from paranasal sinuses [7, 9]. To the best of our knowledge, there have been only a few reports of primary spindle cell carcinomas arising in the orbit. Also, there have been few reports of primary orbital SCC, some of which have occurred following retinal surgery. This has led some others to hypothesize that these lesions arise as a result of implantation of conjunctival epithelial cells, with subsequent malignant degenerations [17, 18].

Some studies have suggested that spindle cell carcinomas either arise as a result of mesenchymal transformation of carcinoma cells or originate from a single pluripotent stem cell that differentiates into epithelial and mesenchymal components [19, 20]. Since the orbit is known to harbor a population of these pluripotent stem cells [21], one could postulate that primary spindle cell carcinomas of the orbit might originate as a result of malignant degeneration of these stem cells into epithelial and mesenchymal components.

Very little is known about the clinical behavior of primary SCC of the orbit, and almost nothing is known about primary orbital spindle cell carcinomas. In addition, the optimal management strategy is also not described due to the small number of reported cases. To the best of our knowledge, this is the one of few known case of primary orbital spindle cell carcinoma [22]. This case is also unique since it describes an atypical presentation of an orbital tumor with limbal ischemia and exudative retinal detachment.

To the date, we did not found any specific mechanism that may relate limbal ischemia to the spindle cell carcinoma. Further cases reports, however, are needed to describe the different clinical presentations and management strategies of this rare clinical entity.

## Data Availability

The datasets used during the current study are available from the corresponding author on reasonable request.

## Abbreviations

SMA: Smooth muscle antigen
CD: Cluster of differentiation
SCC: Squamous cell carcinoma
BCVA: Best corrected visual acuity
CT: Computed tomography
MRI: Magnetic resonance imaging
AIM: Adriamycin + Ifosfamide + Mesna
GI: Gasterointestinal
